# Supplementary material for: The causal role of circulating inflammatory markers in osteoporosis: a bidirectional Mendelian randomized study
Source: Front Immunol. 2024 Jul 18;15:1412298. doi: 10.3389/fimmu.2024.1412298 (PMC11291241; doi:10.3389/fimmu.2024.1412298)
Supplement: Supplementary file 1 [file DataSheet_1.zip › supplementary material/OSTEOPOROSIS-CIM/GCST90274760.tsv.gz_leave_one_out.pdf]

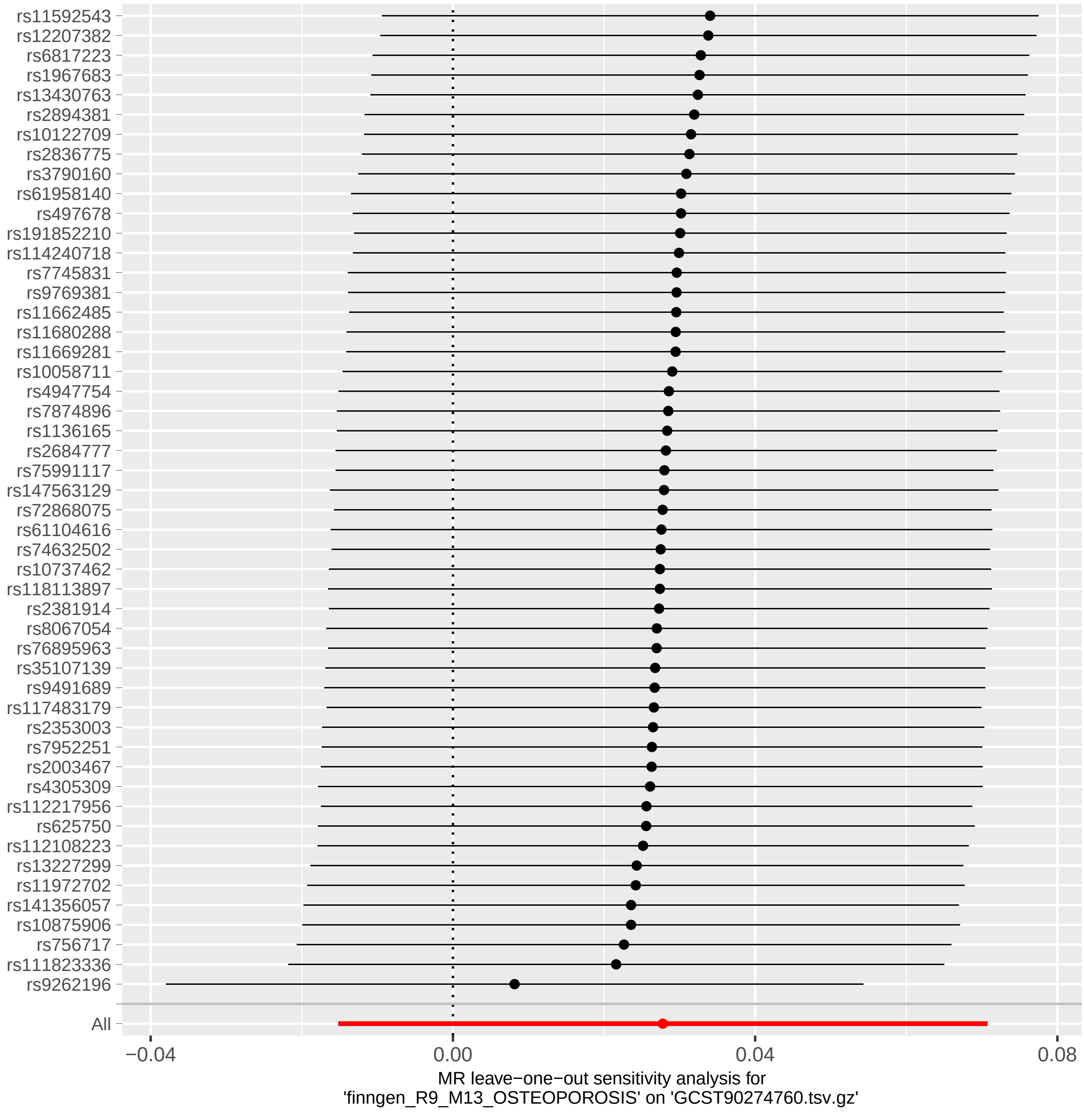

rs11592543
rs12207382
rs6817223
rs1967683
rs13430763
rs2894381
rs10122709
rs2836775
rs3790160
rs61958140
rs497678
rs191852210
rs114240718
rs7745831
rs9769381
rs11662485
rs11680288
rs11669281
rs10058711
rs4947754
rs7874896
rs1136165
rs2684777
rs75991117
rs147563129
rs72868075
rs61104616
rs74632502
rs10737462
rs118113897
rs2381914
rs8067054
rs76895963
rs35107139
rs9491689
rs117483179
rs2353003
rs7952251
rs2003467
rs4305309
rs112217956
rs625750
rs112108223
rs13227299
rs11972702
rs141356057
rs10875906
rs756717
rs111823336
rs9262196
All
−0.04
0.00
0.04
0.08
MR leave−one−out sensitivity analysis for
'finngen_R9_M13_OSTEOPOROSIS' on 'GCST90274760.tsv.gz'
